# The Lived Experiences of Family Members Caring for Pediatric Patients With Extremely Rare Congenital Heart Conditions

**DOI:** 10.7759/cureus.99391

**Published:** 2025-12-16

**Authors:** Matthew J Scearbo, Harvey N Mayrovitz

**Affiliations:** 1 Medicine, Nova Southeastern University Dr. Kiran C. Patel College of Osteopathic Medicine, Tampa, USA; 2 Medical Education, Nova Southeastern University Dr. Kiran C. Patel College of Allopathic Medicine, Davie, USA

**Keywords:** congenital heart disease, psychosocial impact, quality of life, rare conditions, single ventricle defects, survey, total anomalous pulmonary venous return (tapvr), truncus arteriosus

## Abstract

Introduction: Extremely rare congenital heart conditions may differ significantly from common congenital heart conditions in terms of medical management, treatment demands, outcomes, and quality of life, creating unique challenges for parents and families of affected children. Because these conditions are so uncommon, there may be limited information available for parents to understand what to expect. This study aimed to address this gap by surveying caregivers of children with these conditions.

Methods: A 21-item survey was distributed to online support groups for parents of children with rare congenital heart diseases. The survey aimed to collect caregiver-reported, descriptive data on the child's quality of life, medical care experience, and psychological, emotional, and physical functioning, as well as the caregivers' feelings. The results were analyzed and presented in both graphical and numerical formats to provide a summarized report that reflected the experiences of these families.

Results: A total of 448 responses were received, reporting on 23 different extremely rare congenital heart conditions. Three conditions had 50 or more responses and form the main basis of this report: total anomalous pulmonary venous return (TAPVR, N = 90), truncus arteriosus (TA, N = 69), and single-ventricle defects (SVD, N = 58). Children with TAPVR had the highest percentage of independent functioning (60, 67%) and the lowest percentage of developmental delay or physical symptoms (32, 35.8%). Corresponding values for TA were 65.2% (45) and 29% (20), and for SVD were 48.3% (28) and 14.6% (8). Moderate to severe activity restriction was most frequently reported in the SVD group (21, 36.2%), followed by TA (15, 21.7%), and TAPVR (9, 9.9%). Caregivers in all three groups reported distressing emotions at over 70%, with worry and anxiety cited at least 85% of the time. Respondents for TA had the lowest rate of healthcare access difficulty (6, 8.7%), followed by TAPVR (13, 14.5%) and SVD (13, 22.4%). Nearly half of all respondents reported economic hardship, with TAPVR reporting the highest burden (48, 53%), and TA and SVD having nearly identical rates of 43.5% (30) and 43.1% (25), respectively.

Conclusions: The survey results highlight the unpredictability that the future may hold for parents of children with these rare conditions. Thankfully, the results suggest that with appropriate medical interventions and support, many of these children can become fully functional without facing a significant healthcare burden. For those with less favorable outcomes, the survey results help shed light on the potential challenges parents may encounter. Early education is vital in preparing parents for the road ahead and enabling them to provide the best support for their child.

## Introduction

An extremely rare congenital heart disease is defined as one with an incidence of one in 10,000 or less [1]. Parents faced with the reality of having a child diagnosed with such a rare disease may find it challenging, intimidating, and distressing. Due to the disease's rarity, some or perhaps many parents may not have a clear understanding of what to expect regarding their child's medical care or quality of life. The primary goal of this study was to provide families of children diagnosed with extremely rare heart conditions with helpful information specific to their condition. Parents who are currently or have already endured this journey were surveyed to identify their child's level of restriction, quality of life, and degree of healthcare burden. In addition, the study aimed to identify the emotions that parents may feel during this experience.

This is an exploratory study that, via survey, aims to provide insight on the experiences of family members caring for pediatric patients with one of the extremely rare congenital heart diseases. The survey was designed to collect information about the child's quality of life, medical care experience, and psychological, emotional, and physical well-being. Although 23 extremely rare pediatric heart conditions were initially identified, only three of these conditions had 50 or more responses. The threshold for the number of responses for a condition to be included in this paper was set pragmatically at 50 responses to avoid reporting misleading or inconclusive data due to small sample sizes. One of these conditions was total anomalous pulmonary venous return (TAPVR). In this condition, the oxygenated blood exiting the lungs, which normally flows to the left atrium, is instead diverted to the right atrium, the venous system, or forms other abnormal connections [2]. Another condition with 50 or more responses was truncus arteriosus. This condition is characterized by a single arterial trunk supplying the systemic, pulmonary, and coronary circulations [3]. The third condition, having adequate responses, was related to children born with a single-ventricle heart defect [4,5].

The current literature addresses quality of life in congenital heart conditions collectively, with an emphasis on adulthood [6-8]. There is limited information available specifically on the quality of life of pediatric patients with TAPVR, a gap this study addresses. Quality of life in truncus arteriosus is addressed in the current literature, but mainly focuses on adults [9,10]. One study from 2013 addresses morbidity following surgical repair of truncus arteriosus; the present study extends existing data, which is needed as procedures for these conditions continue to become more successful [11]. There is also data on the quality of life of single-ventricle patients, and the present study contributes to that body of work [12-14].

Healthcare utilization for those with congenital heart conditions is addressed in the literature, with data reflecting all congenital heart conditions [15-17]. An existing study examines the healthcare burden over time during surgical correction in single-ventricle patients [18]. This survey helps address healthcare use throughout the child’s life, including the surgical period. There is limited information available on healthcare utilization and burden for pediatric patients with TAPVR or truncus arteriosus; hence, the present study provides valuable information for parents of children with these conditions.

Existing literature does address traumatic stress, psychiatric, and psychological impact associated with congenital heart conditions [19-24]. However, the majority of existing literature provides an overview of the emotional journey across all congenital heart conditions. The present study contributes to the existing literature and further supports the need for accessible mental health resources for these parents, as identified in the current literature. Most importantly, the present study highlights the emotional journey of parents of children with these three rare conditions, making it highly insightful and directly applicable to parents of children with these conditions.

It is important to consider that all congenital heart conditions vary significantly, with common conditions such as atrial or ventricular septal defects often being entirely asymptomatic with little effect on the patient’s life. The rare conditions discussed in this paper differ significantly from many other common congenital cardiac conditions. Therefore, the majority of existing literature on congenital heart conditions may not be as insightful to families as the data presented here. Having information that is specific to their child’s condition, coming directly from parents who have undergone a similar experience, is crucial for parents to have readily available, underscoring the need for this survey. It is hoped that the information gathered and reported here, based on survey feedback, will be valuable to parents currently facing, or who will face, this type of life experience and will better prepare them for the future regarding their child's health and quality of life.

## Materials and methods

Participants and data acquisition

The data from this study were obtained via a survey from parents of children who had been diagnosed as having a rare congenital heart condition. For this purpose, an anonymous survey consisting of 21 questions was created. Questions for the survey were written and reviewed by the authors based on what they believed would be valuable information for caregivers that is currently unaddressed in the literature; hence, the survey was not tested or validated. There were no validated questionnaires available that the authors felt specifically addressed all aspects of the questions they were looking to address; hence, a self-made, non-validated survey was used. Responses to the survey were obtained during the four-month interval from December 9, 2021, to April 10, 2022. Demographics were not collected. With the limited availability of respondents for these rare conditions, further stratifying results by demographic would not have contributed to the conclusions drawn. For these rare diseases where data is limited, the information the survey provides is valuable regardless of the respondent demographics. To obtain informed consent, the survey included a participation letter that named the individuals conducting the study and explained the purpose of the research project, which focused on extremely rare congenital heart conditions. Facebook was used to disseminate the survey to 20 support groups with their permission. These groups were the following: Congenital Heart Defect Awareness: Truncus Arteriosus, CCTGA Parents Forum, Oculofaciocardiodental Syndrome, Syndromes Andersen Tawil Syndromes Maladie Rare, Shone’s Complex Ohio, Congenital Heart Disease, CHARGE Syndrome - Florida Friends!, CHD & Heterotaxy Syndrome Info and Support, Shone’s Complex, CFC Syndrome, CHARGE Syndrome - Members only, DORV Heart Defect, Cantu Syndrome Support Group, Pulmonary Vein Stenosis, Truncus Arteriosus Kids and Adults, Ebstein’s Anomaly, Double Inlet Left Ventricle Support, Children with Congenital Heart Defects, CHARGE Syndrome, and Aortic Valve Replacement Group. The survey was posted with permission on websites to reach support groups for 23 identified congenital heart conditions. It was encouraged that only one parent or primary caregiver per child with a heart defect filled out the survey. The survey and the research study was approved by the Institutional Review Board of Nova Southeastern University Dr. Kiran C. Patel College of Allopathic Medicine, Davie, USA (IRB #2021-574, dated: December 7, 2021). Although survey responses from parents for each of these conditions were received, only the three previously mentioned received 50 or more responses.

Survey questions and choices

The questions and answer choices included in the survey are listed in Table 1.

**Table 1:** Survey questions and answer choices The survey had 21 total questions, which are listed with their answers in table.

#	Survey Question	Answer Choices
1	What congenital disease was the child diagnosed with?	Open choice
2	What is the sex of the child?	Open choice
3	What was the child’s age at their diagnosis?	Prenatal, Less than a week, One week, Two weeks, Three weeks, One month, Three months
4	What was the year that the child was diagnosed?	Open choice
5	Is the child still being treated for the condition?	Yes, No
6	What is the degree to which the child’s condition challenges their function?	Requires no assistance, Requires some assistance, Requires a lot of assistance
7	What is your confidence level that these challenges will be reduced in the future?	No confidence, Low confidence, Medium confidence, High confidence
8	How often does the child need to visit a medical provider about their condition?	Every month, Every three months, Every six months, Annually
9	Regarding the child’s participation in age-appropriate physical activities, what amount of restriction does their condition cause them to have?	None, Mild, Moderate, Severe
10	What was the child’s approximate longest hospital stay for their condition?	One week, Two weeks, Three weeks, One month, Two months, More than two months
11	Since the diagnosis, how often has the child been hospitalized for their condition?	Open choice
12	What was the approximate length of time between the child’s first signs or symptoms and receiving the correct diagnosis?	Diagnosed immediately, Diagnosed in < one month, Diagnosed in one to three months, Diagnosed in three to six months, Diagnosed in more than six months
13	How was the experience of finding appropriate medical care, including a qualified specialist?	Extremely easy, Somewhat easy, Neither easy nor difficult, Somewhat difficult, Extremely difficult
14	Did your family experience a significant economic hardship due to the child’s diagnosis?	Yes, No
15	How old was the child at the time of the first heart surgical intervention?	Less than a week, One week, Two weeks, Three weeks, One month, Two months, Three months, More than three months
16	How many surgical procedures did the child have?	Open choice
17	How many different daily medications is the child currently on due to their condition?	Open choice
18	Did you or any other parent or guardian in your family experience any of the following as a result of the child’s medical diagnosis? (Choose all that apply)	Anger, Anxiety, Fear, Guilt, Helplessness, Sorrow, Worry
19	If the child is between 0-5 years, do you agree or strongly agree that, due to the child’s condition, the following apply to the child? (Choose all that apply)	Gets special treatment from adults, Physical development delay, Motor development delay, Speech/language development delay, Behavior is difficult to manage, Tires easily, Feels sluggish or in pain, Has sleeping problems, Medicine causes side effects, Can’t eat or drink everything desired, None of the above
20	If the child is between 6-12 years, do you agree or strongly agree that, due to the child’s condition, the following apply to the child? (Choose all that apply)	Gets special treatment from adults, Tires easily, Difficulty with homework, Difficulty with schoolwork, Hard time making friends, Feels different from others, Afraid of dying or of the future, Misses social activities, Is self-destructive or angry, None of the above
21	If the child is between 13-18 years, do you agree or strongly agree that, due to the child’s condition, the following apply to the child? (Choose all that apply)	Gets special treatment from adults, Tires easily, Difficulty with homework, Difficulty with schoolwork, Hard time making friends, Feels different from others, Afraid of dying or of the future, Misses social activities, Is self-destructive or angry, None of the above

Data analysis

Responses were stratified by the type of congenital heart condition that the respondent’s child had and analyzed and presented via graphics and tabular data. For each of the reported conditions, the distribution of responses to the survey questions was calculated and presented mostly in graphical format, with other data in table format. Distributions for all graphics and other calculations were determined using Excel (Microsoft Excel, Baton Rouge, LA). No statistical analysis was done as such were not relevant to the study goals.

## Results

Distribution of responses

There were 23 extremely rare congenital pediatric conditions that were initially identified and for which responses were received. These are listed in Table 2, along with the number of responses for each condition. The total number of responses was 448. As noted in the Introduction, it was deemed necessary to achieve at least 50 responses to provide a meaningful and detailed examination and reporting. In the present case, this criterion was met by three congenital conditions: total anomalous pulmonary venous return (TAPVR), truncus arteriosus (TA), and single-ventricle defects (SVD). These three extremely rare cardiac congenital conditions, together, accounted for 48.4% of the total responses, with the remainder of the responses spread across 20 other conditions.

**Table 2:** Response distribution There were 23 congenital conditions for which responses were received. The number of responses for each is listed in the second column, and the percentage of the total responses (448) is listed in the third column. CHARGE: Coloboma, Heart defects, Atresia choanae, Retarded growth/development, Genital abnormalities, and Ear anomalies.

Congenital Condition	Number	Percent
Total anomalous pulmonary venous return (TAPVR)	90	20.1
Truncus arteriosus (TA)	69	15.4
Single-ventricle defects (double-inlet left ventricle)	58	12.9
Shone's complex	42	9.4
Ebstein anomaly	31	6.9
Holt-Oram syndrome	22	4.9
Heterotaxy syndrome	22	4.9
Congenital pulmonary vein stenosis	21	4.7
CHARGE syndrome	16	3.6
MED13L syndrome	14	3.1
Alagille syndrome	13	2.9
Andersen-Tawil syndrome	10	2.2
Congenitally corrected/levo-transposition of the great arteries (ccTGA/L-TGA)	8	1.8
Double-outlet right ventricle	6	1.3
Cardiofaciocutaneous syndrome	6	1.3
Congenital complete heart block	4	0.9
Cantú syndrome	4	0.9
Pulmonary atresia with intact ventricular septum	3	0.7
Alström syndrome	3	0.7
Oculofaciocardiodental syndrome	2	0.4
Congenital mitral valve anomalies	2	0.4
Char syndrome	1	0.2
Arterial tortuosity syndrome	1	0.2

Total anomalous pulmonary venous return (TAPVR)

**Figure 1:**
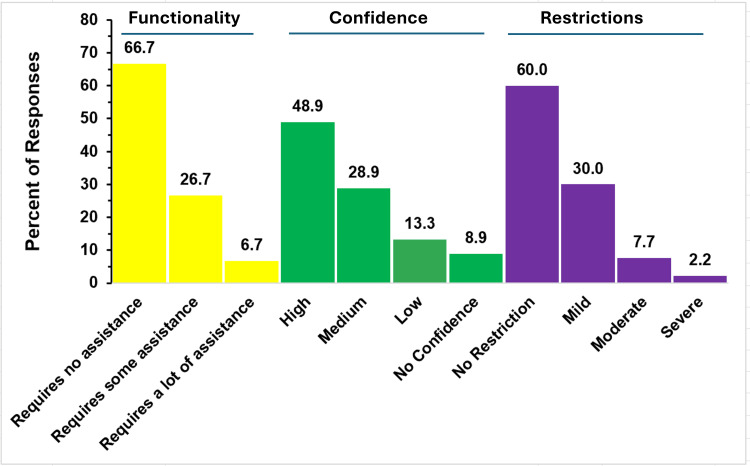
Functionality and restrictions in TAPVR The figure shows data from respondents who are parents of children with total anomalous pulmonary venous return (TAPVR). Percentages are based on the total number of TAPVR respondents (N = 90) who answered the three questions regarding functionality, restrictions, and confidence that challenges may be reduced in the future.

A total of 90 responses from parents of children with TAPVR were received and processed. Figure 1 summarizes their responses to questions related to the child’s functionality, parent confidence in improvement, and condition-related restrictions.

Assistance Required by the Child

Of the 90 respondents, 60 (66.7%) reported that their child required no assistance with daily activities and was independently functioning. Twenty-four respondents (26.7%) indicated that their child needed some assistance, whereas only six (6.7%) reported that their child required significant help with functioning.

Parents’ Confidence in Improvement

When asked about their confidence that their child’s functionality would improve, approximately half (44, 48.9%) were highly confident, and more than three-quarters (70, 77.8%) of responders indicated high or medium confidence. However, nearly a quarter of the respondents (20, 22.2%) expressed low or no confidence in improvement.

Child’s Level of Activity Restrictions

Parents were asked to classify their child’s level of activity restriction. Out of 90, 54 parents (60%) reported that their child has no activity restrictions. Mild restriction was chosen by 27 (30%) responders. Just seven responders (7.7%) reported that their child has moderate activity restriction, and only two (2.2%) reported that their restriction was severe.

Child Developmental and Physical Challenges

**Figure 2:**
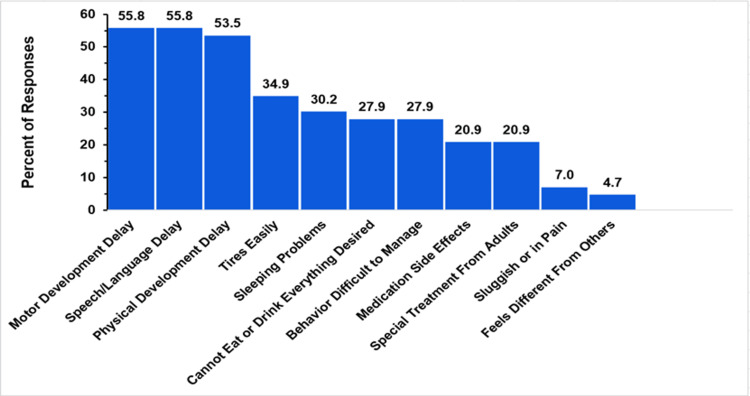
TAPVR development delays and symptoms The figure shows data from respondents who are parents of children with total anomalous pulmonary venous return (TAPVR). Percentages are based on the total number of TAPVR respondents who selected at least one of the symptoms or development delays (N = 43). Respondents could not select an “all of the above” option.

Figure 2 summarizes response percentages about child developmental and physical challenges experienced by children with TAPVR, as reported by their parents. Multiple choices could be chosen in response to this question, but there was no option to select “all of the above.”

Of the 90 overall respondents, 67 responded to this aspect. Approximately a third (35.8%) stated that none of the child development choices provided in the survey were present in their child's survey. For the remaining 43 responders, the distribution of responses is detailed in Figure 2. For these responders, 55.8% (24 respondents) reported motor, speech, and language delays. In addition, 23 children (53.4%) were noted to have physical developmental delays. Other commonly reported issues were tiring easily (15, 34.9%) and sleeping difficulties (13, 30.2%). Behavioral issues and eating or drinking restrictions were each reported by 12 respondents (27.9%). Getting special treatment from adults or dealing with medication side effects were reported by nine respondents each (20.9%). Uncommon problems included feeling sluggish or in pain (3, 7%) and feeling different from others (2, 4.7%).

Caregiver Emotional Burden

**Figure 3:**
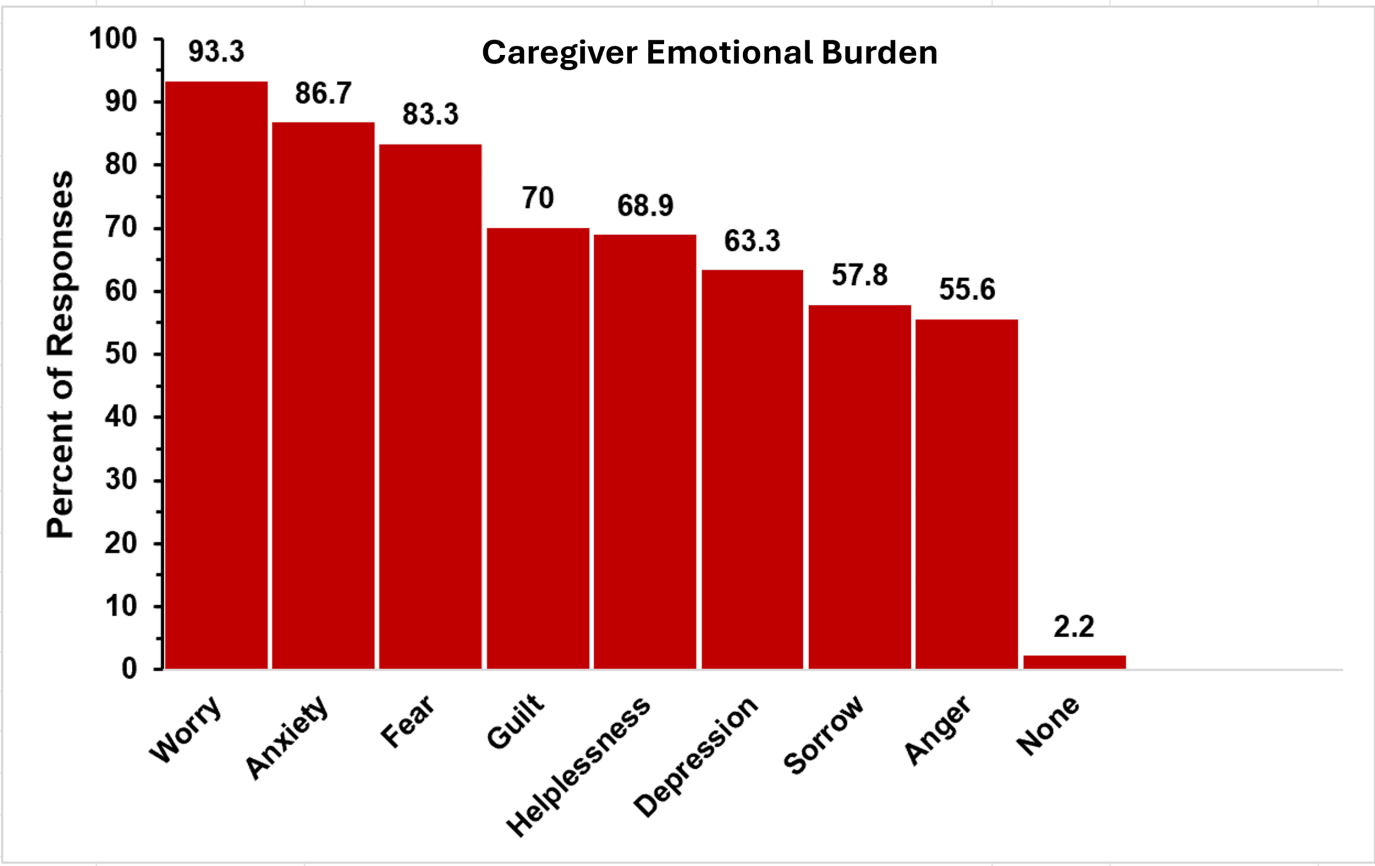
Parent feelings in TAPVR The figure shows data from respondents who are parents of children with total anomalous pulmonary venous return (TAPVR). Percentages are based on the total number of TAPVR respondents (N = 90) who answered the question regarding feelings they have experienced as a product of their child’s condition. Respondents could not select an “all of the above” option.

The emotional burden on caregivers was significant, as indicated by survey results, as depicted in Figure 3. Multiple choices could be chosen in response to this question, but there was no option to select “all of the above.”

Of the 90 responding to this issue, the most frequently reported feeling was worry, cited by 84 respondents (93.3%), with anxiety (78, 86.7%), fear (75, 83.3%), and guilt (63, 70.0%) following closely behind. Many parents also expressed sadness (55, 61.1%), helplessness (53, 58.9%), and grief (49, 54.4%). The results indicate that nearly all parents of children with TAPVR experience some distress, with only 2.2% (2 respondents) claiming they felt none of the above emotions.

Access to Care and Economic Hardship of Caregivers

**Figure 4:**
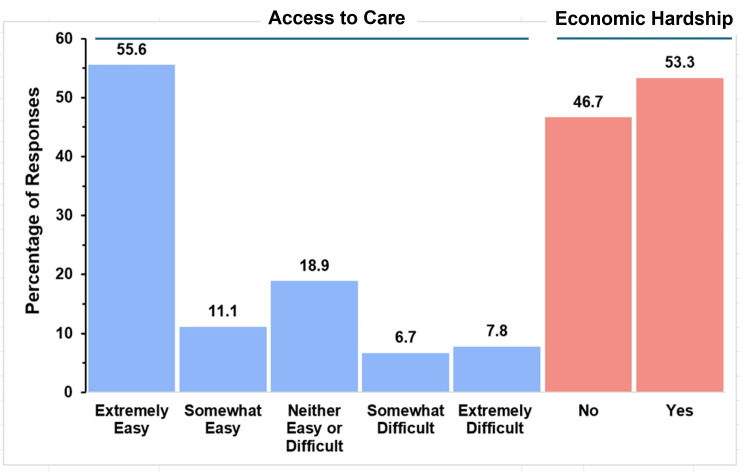
Healthcare access and financial burden in TAPVR The figure shows data from respondents who are parents of children with total anomalous pulmonary venous return (TAPVR). Percentages are based on the number of TAPVR respondents (N = 90) who answered the questions regarding access to care and economic hardship.

Figure 4 graphically summarizes the responses to the survey questions investigating this aspect for the parents of children with TAPVR.

Parents were asked about how easy it was to access care for a child with TAPVR and whether or not that care caused them economic hardship. The majority of respondents (50, 55.6%) found it extremely easy to access care, and 10 (11.1%) reported it was somewhat easy. The second most common response (70, 18.9%) was that care was neither easy nor difficult to access. Only 13 (14.4%) respondents reported that care was difficult to access, with 6.7% (6 respondents) indicating that it was somewhat difficult and 7.8% (7 respondents) indicating it was extremely difficult. Economic hardship was nearly an even split, with a narrow majority (48, 53.3%) experiencing significant financial difficulties.

Hospital Admissions and Number of Surgeries

**Figure 5:**
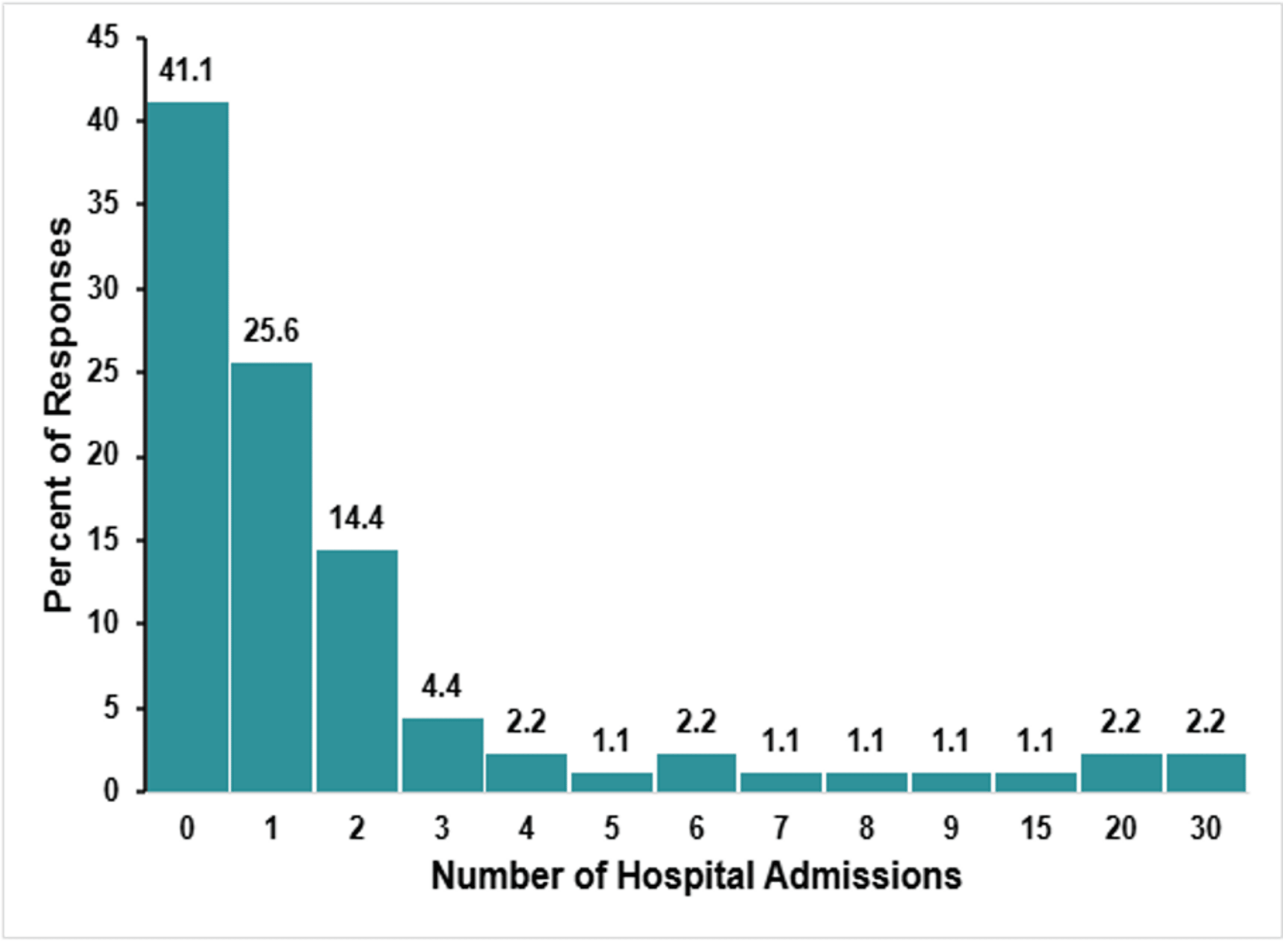
TAPVR hospitalizations The figure shows data from respondents who are parents of children with total anomalous pulmonary venous return (TAPVR). Percentages are based on the number of TAPAVR respondents (N = 90) who answered the question regarding the number of hospitalizations their child has needed.

Figure 5 illustrates the number of hospital admissions the child experienced.

**Figure 6:**
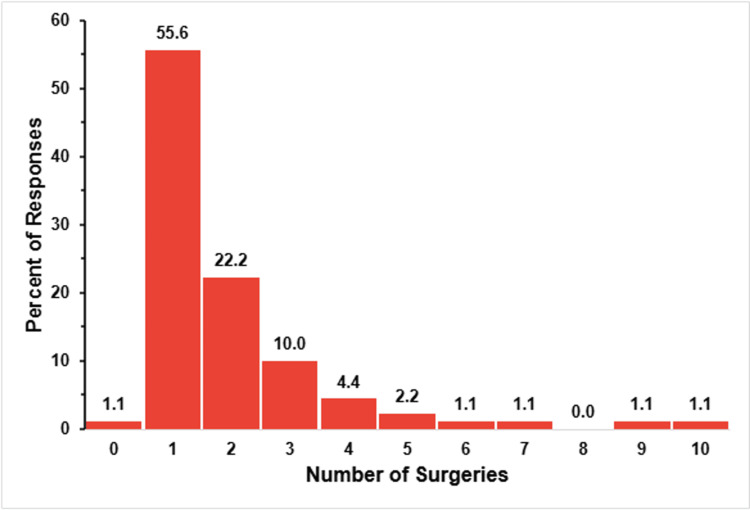
TAPVR surgeries The figure shows data from respondents who are parents of children with total anomalous pulmonary venous return (TAPVR). Percentages are based on the number of TAPVR respondents (N = 90) who answered the question regarding the number of surgeries their child has needed.

Figure 6 illustrates the number of surgeries that respondents indicated their child underwent for the congenital cardiac condition.

The overall hospitalization burden of this group was low. Many respondents (37, 41.1%) reported no prior hospital admissions, while 25.6% (23 respondents) had one admission, 14.4% (13 respondents) reported two admissions, and 4.4% (four respondents) reported three. A total of 14.4% (13 respondents) of respondents reported their child had more than three admissions, with some having very frequent visits. Most of them (nine, 9.9%) reported between four and nine admissions. An extensive burden was reported in only one respondent (1.1%), who had 15 admissions, and two respondents (2.2%) each reported 15 and 30 admissions, respectively.

Every respondent, except one (1.1%), indicated that their child had undergone at least one surgery. The most frequently reported number of surgeries was one (50, 55.6%) followed by 22.2% (20 respondents) reporting two surgeries, and 10.0% (nine respondents) reporting three surgeries. Few reported a high surgical burden, including 4.4% (four respondents) with four surgeries, 2.2% (two respondents) with five surgeries, and just one respondent each reporting six, seven, nine, or 10 surgeries.

Other Relevant Survey Data

Table 3 indicates additional findings as reported in the survey.

**Table 3:** Response distribution for remaining TAPVR questions (N = 90) The response distribution for the following questions regarding TAPVR was not displayed in graphic format, but is included in table: age at diagnosis, medical visit frequency, medications needed for condition, and age at first surgery. Percentages are based on the total number of TAPVR respondents. TAPVR: total anomalous pulmonary venous return.

Age at Diagnosis	N (%)	Medical Visit Frequency	N (%)	Medications Needed for Condition	N (%)	Age at First Surgery	N (%)
Prenatal	7 (8%)	Never	2 (2%)	0	61 (68%)	< 1 week	42 (47%)
< 1 week	60 (67%)	Weekly	1 (1%)	1	10 (11%)	1 week	15 (17%)
1 week	6 (7%)	Monthly	6 (7%)	2	6 (7%)	2 weeks	9 (10%)
2 weeks	2 (2%)	3 months	15 (17%)	3	6 (7%)	3 weeks	6 (7%)
3 weeks	4 (4%)	6 months	17 (19%)	4	2 (2%)	1 month	6 (7%)
1 month	4 (4%)	Yearly	49 (54%)	6	2 (2%)	2 months	4 (4%)
2 months	2 (2%)			7	1 (1%)	3 months	4 (4%)
3 months	2 (2%)			10	2 (2%)	4 months	2 (2%)
4 months	2 (2%)					12 months	1 (1%)
12 months	1 (1%)					13 years	1 (1%)
13 years	1 (1%)						

Most children with TAPVR (60, 67%) were diagnosed with their condition within the first week of life, with an additional 8% (seven respondents) diagnosed prenatally and 8% (seven respondents) between one and two weeks of age. Only 16% (15 respondents) were diagnosed beyond two weeks of age. One child (1.1%) was diagnosed as late as 13 years. Most families (49, 54%) reported having yearly medical follow-up visits. Others reported visits every six months (17, 19%), every three months (15, 17%), and monthly (six, 7%). Just one respondent (1.1%) reported their child needed weekly visits, and only two respondents (2.2%) reported that they do not have a follow-up at all. Approximately two-thirds of respondents (61, 68%) indicated that their child did not require any medications for their condition, whereas 25% (22 respondents) of the children were taking from one to three medications for their condition. Only 7% (six respondents) were taking more medications than this. Almost half of the children (42, 47%) had surgery within the first week of life. An additional 17% (15 respondents) had surgery at one week, 10% (nine respondents) at two weeks, with 88% (78 respondents) having had surgery within the first month of life.

Truncus arteriosus

**Figure 7:**
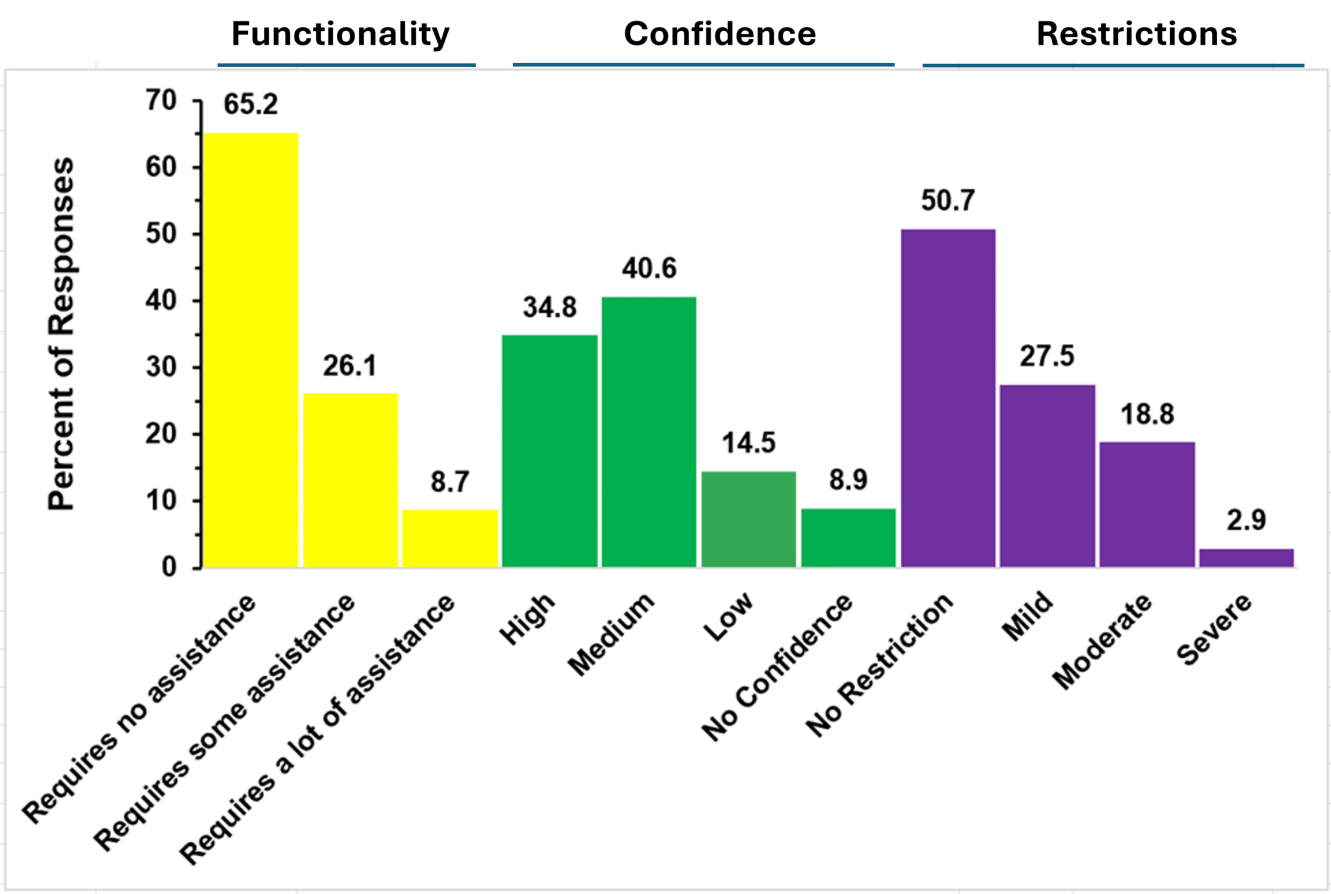
Functionality and restrictions in truncus arteriosus The figure shows data from respondents who are parents of children with truncus arteriosus. Percentages are based on the total number of truncus arteriosus respondents (N = 69) who answered the three questions regarding functionality, restrictions, and confidence that challenges may be reduced in the future.

A total of 69 responses from parents of children with truncus arteriosus were received. Their responses to their child’s functionality and restrictions are shown in Figure 7.

Assistance Required

Almost two-thirds of parents (45, 65.2%) reported that their child requires no assistance at all, and 26.1% (18 respondents) indicated their child requires some assistance. Just 8.7% (six respondents) said their child requires a lot of assistance.

Parents’ Confidence in Improvement

Slightly more than three-quarters of parents (53, 76.8%) reported at least medium or high confidence that their child’s restrictions would improve. Out of the remaining respondents, 14.5% (10 respondents) reported low confidence, and 8.7% (six respondents) reported having no confidence.

Child’s Level of Activity Restrictions

Approximately half of the respondents (35, 50.7%) reported that their child has no activity restrictions. A little more than a quarter (19, 27.5%) reported mild activity restrictions. A total of 13 respondents (18.8%) reported moderate restriction, and just 2.9% (two respondents) reported severe restrictions.

Child Developmental and Physical Challenges

**Figure 8:**
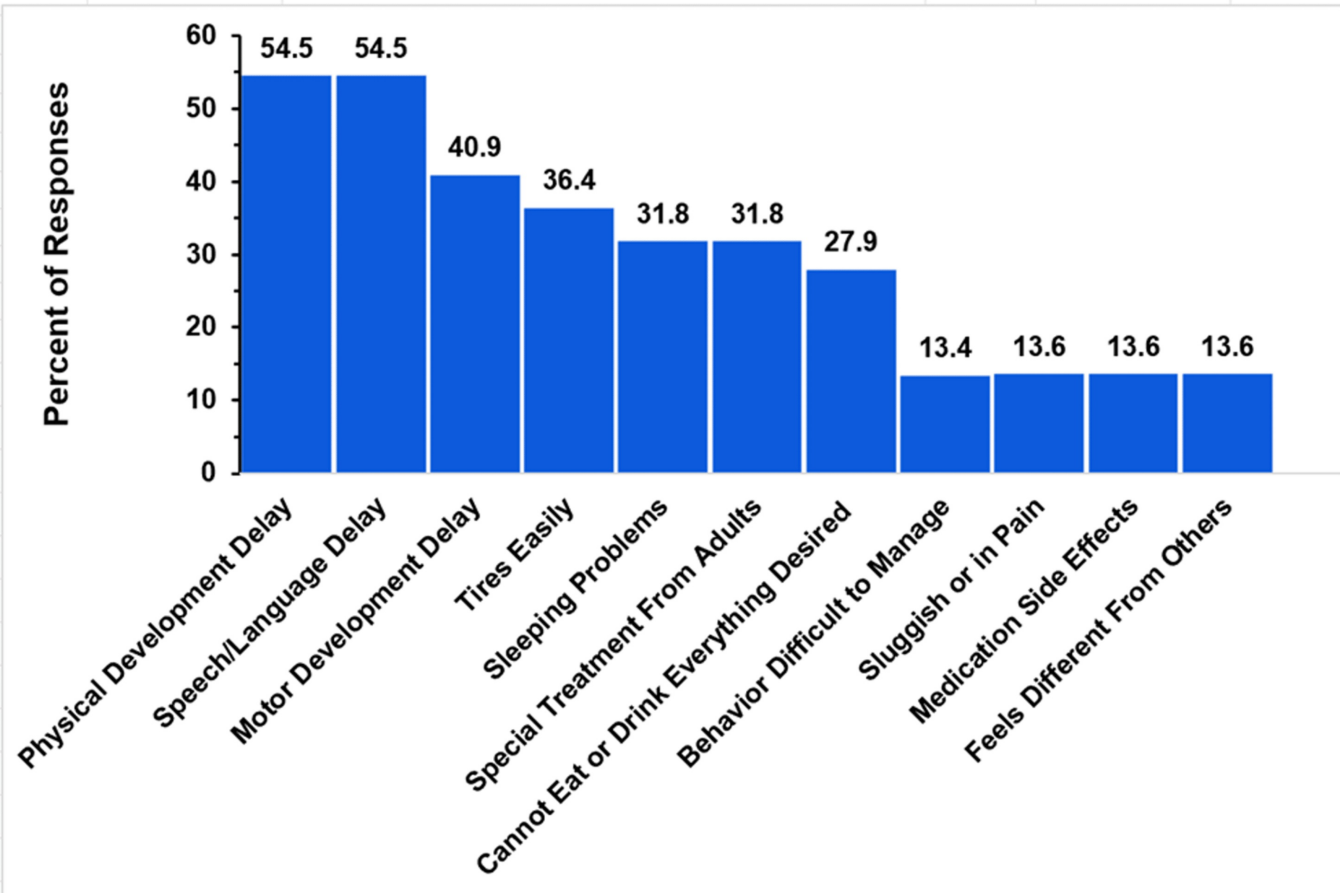
Truncus arteriosus development delays and symptoms The figure shows data from respondents who are parents of children with truncus arteriosus. Percentages are based on the total number of truncus arteriosus respondents (N = 69) who reported one of the listed symptoms of developmental delays (N = 22). Respondents could not select an “all of the above” option.

Figure 8 demonstrates the percentage of respondents who experienced certain developmental delays and physical challenges. Multiple choices could be chosen in response to this question, but there was no option to select “all of the above.”

Of the 69 respondents, only 31 (45%) answered this question. Of the 31, nine (29%) selected “none of the above” for developmental and physical challenges. For the remaining 22 respondents who did indicate an issue, the distribution of developmental and physical issues is shown in Figure 8. This distribution is based on these 22 responses. The most frequently reported issues were physical developmental delay and speech/language developmental delay, which were each reported by 12 respondents (54.5%). Motor developmental delay was reported by nine respondents (40.9%). Other reported issues included tiring easily (eight, 36.4%), sleep problems, and receiving special treatment from adults (seven, 31.8%), as well as an inability to eat or drink everything desired (six, 27.9%). The remaining issues cited by three respondents each (three, 13.6%) were behavior that is difficult to manage, sluggishness or pain, side effects from medication, and feeling different from others.

Caregiver Emotional Burden

**Figure 9:**
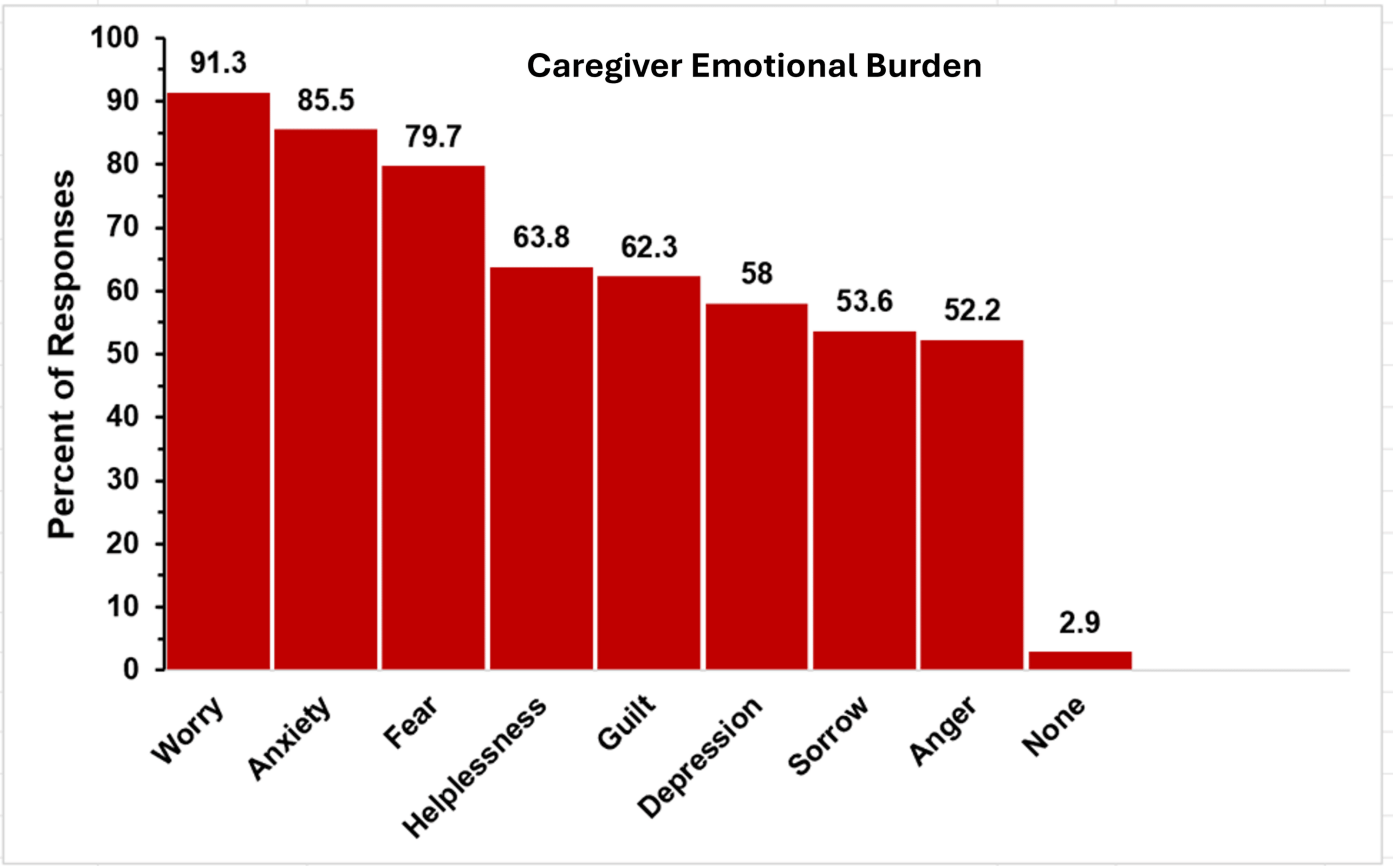
Parent feelings in truncus arteriosus The figure shows data from respondents who are parents of children with truncus arteriosus. Percentages are based on the total number of truncus arteriosus respondents (N = 69) who answered the question regarding feelings they have experienced as a product of their child’s condition. Respondents could not select an “all of the above” option.

The emotional burden on parents of children with truncus arteriosus is shown in Figure 9. Multiple choices could be chosen in response to this question, but there was no option to select “all of the above.”

Parent Feelings

Parents were asked to report the feelings they experience as a result of their child’s truncus arteriosus. The top three most commonly reported emotions were worry, cited by 63 respondents (91.3%), followed closely by anxiety (59, 85.5%) and fear (54, 79.7%). Other commonly reported feelings were helplessness (44, 63.8%), guilt (43, 62.3%), and depression (40, 58.0%). More than half of respondents reported sorrow (37, 53.6%) and anger (36, 52.2%).

Access to Care and Economic Hardship of Caregivers

**Figure 10:**
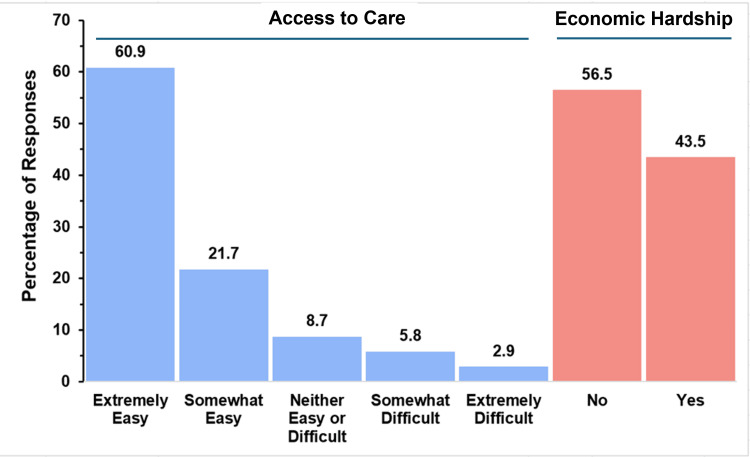
Healthcare access and financial burden in truncus arteriosus The figure shows data from respondents who are parents of children with truncus arteriosus. Percentages are based on the number of truncus arteriosus respondents (N = 69) who answered the questions regarding access to care and economic hardship.

Figure 10 demonstrates the percentages of respondents who faced difficulty accessing care or economic hardships as a result of their child’s care.

In terms of care accessibility, the majority of parents (42, 60.9%) reported that accessing care was extremely easy, and 21.7% (15 respondents) found it somewhat easy. Only 8.7% of respondents (six respondents) reported that accessing care was either somewhat or extremely difficult for them. In terms of economic hardship, 43.5% (30 respondents) reported experiencing financial strain.

Hospital Admissions and Number of Surgeries

**Figure 11:**
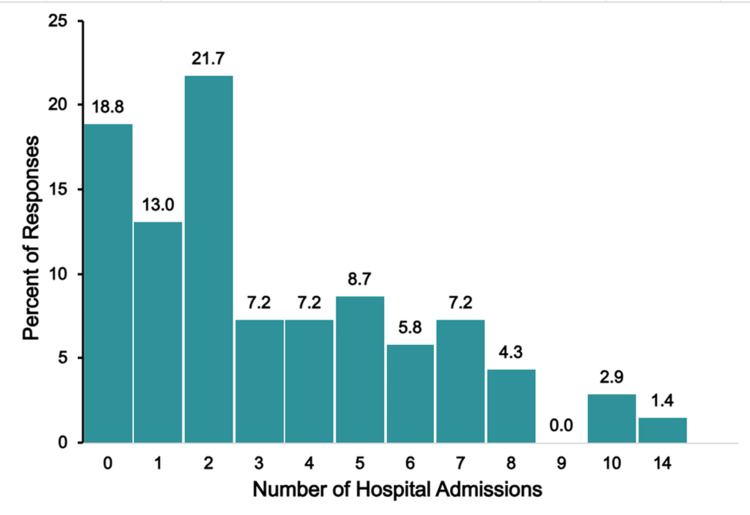
Truncus arteriosus hospitalizations The figure shows data from respondents who are parents of children with truncus arteriosus. Percentages are based on the number of truncus arteriosus respondents (N = 69) who answered the question regarding the number of hospitalizations their child has needed.

Figure 11 shows the number of hospital admissions reported for children with truncus arteriosus.

**Figure 12:**
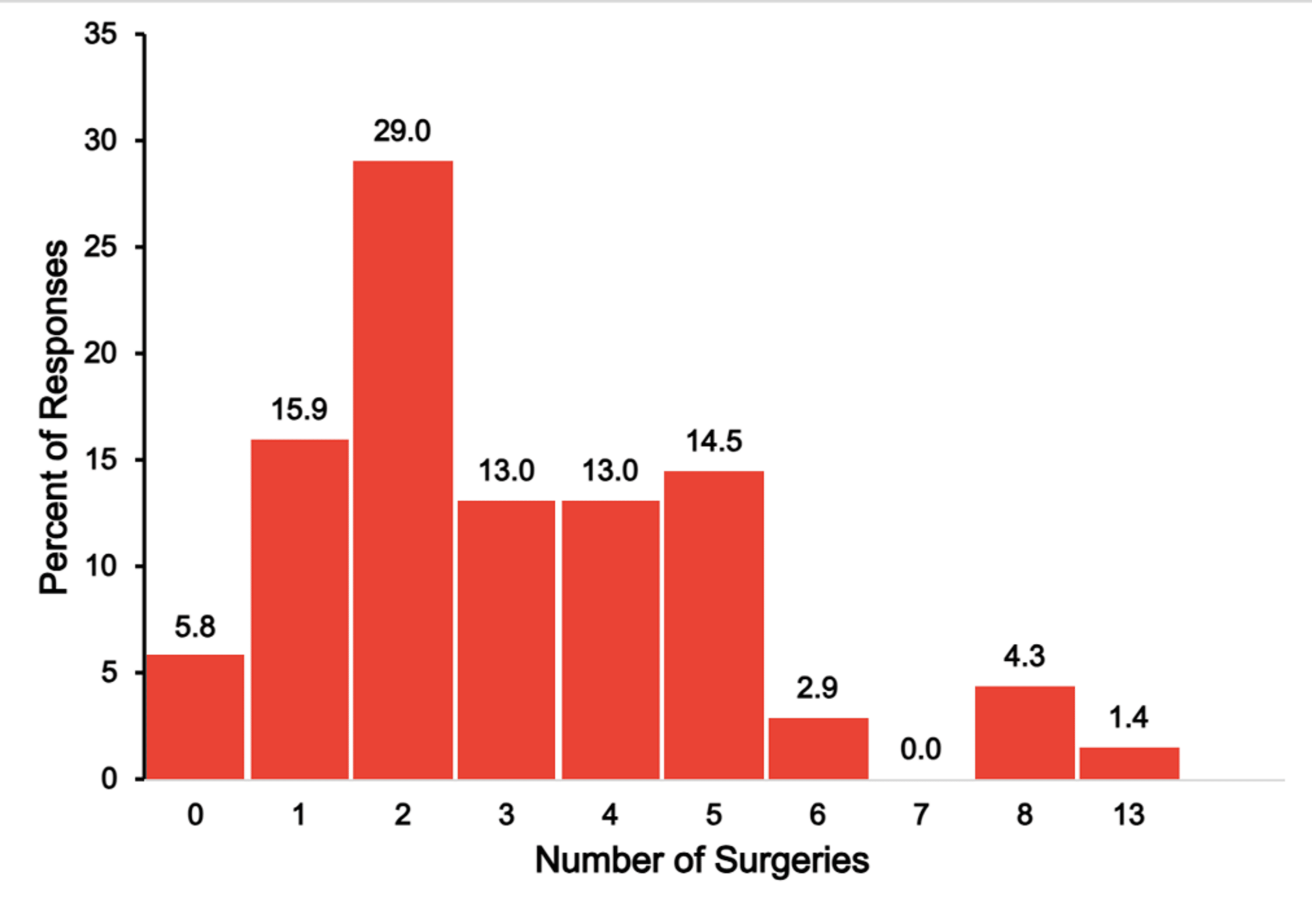
Truncus arteriosus surgeries The figure shows data from respondents who are parents of children with truncus arteriosus. Percentages are based on the number of truncus arteriosus respondents (N = 69) who answered the question regarding the number of surgeries their child has needed.

Figure 12 shows the number of surgeries reported for children with truncus arteriosus.

More than half (37, 53.5%) of respondents reported two or fewer hospitalizations, with 18.8% (13 respondents) reporting none. However, approximately one-fifth of respondents (15, 21.6%) reported six or more hospitalizations, with a small percentage (three, 4.3%) reporting 10 or more. As to the number of surgeries, a wide range of surgical experiences was reported, ranging from none (4, 5.8%) to five or more (16, 23.1%). Two surgeries were the most reported, cited by 29% of respondents (20 respondents).

Other Relevant Survey Data

Table 4 indicates additional findings as reported in the survey.

**Table 4:** Response distribution for remaining truncus arteriosus questions (N = 69) The response distribution for the following questions regarding truncus arteriosus was not displayed in graphic format, but is included in table: Age at diagnosis, medical visit frequency, medications needed for condition, and age at first surgery. Percentages are based on the total number of truncus arteriosus respondents.

Age at Diagnosis	N (%)	Medical Visit Frequency	N (%)	Medications Needed for Condition	N (%)	Age at First Surgery	N (%)
Prenatal	24 (35%)	Never	3 (4%)	0	26 (38%)	< 1 week	21 (30%)
< 1 week	38 (55%)	Weekly	1 (1%)	1	23 (33%)	1 week	17 (25%)
1 month	1 (1%)	Monthly	52 (75%)	2	12 (17%)	2 weeks	11 (16%)
1-6 months	3 (4%)	Yearly months	14 (20%)	3	3 (4%)	3 weeks	4 (6%)
6-12 months	3 (4%)			4	1 (1%)	1 month	7 (10%)
Over one year month	1 (1%)			5	1 (1%)	2 months	1 (1%)
				6	1 (1%)	3 months	4 (6%)
				10	2 (3%)	5 months	1 (1%)
						No surgery months	3 (3%)

Many of the children in this group were diagnosed before birth, with 35% (24 respondents) reporting a prenatal diagnosis. The most common diagnosis was within the first week of life, reported by 55% of respondents (38 respondents), and only 10% of respondents (eight respondents) reported a diagnosis beyond this time. Regarding medical follow-up frequency, three-quarters of respondents (52, 75%) reported visiting their doctor monthly. Annual visits were reported by 20% of respondents (14 respondents), and only 4% (three respondents) reported no medical visits. No medication use was reported by 38% of respondents (26 respondents) in this group. Approximately one-third of the children (23, 33%) required only one medication for their condition, and 17% (12 respondents) required two medications. The most common timing of the first surgery was when the child was less than one week of age, reported by 30% of respondents (21 respondents). First surgeries occurred within the first two weeks of age in 71% of reported cases (49 respondents).

Single ventricle

**Figure 13:**
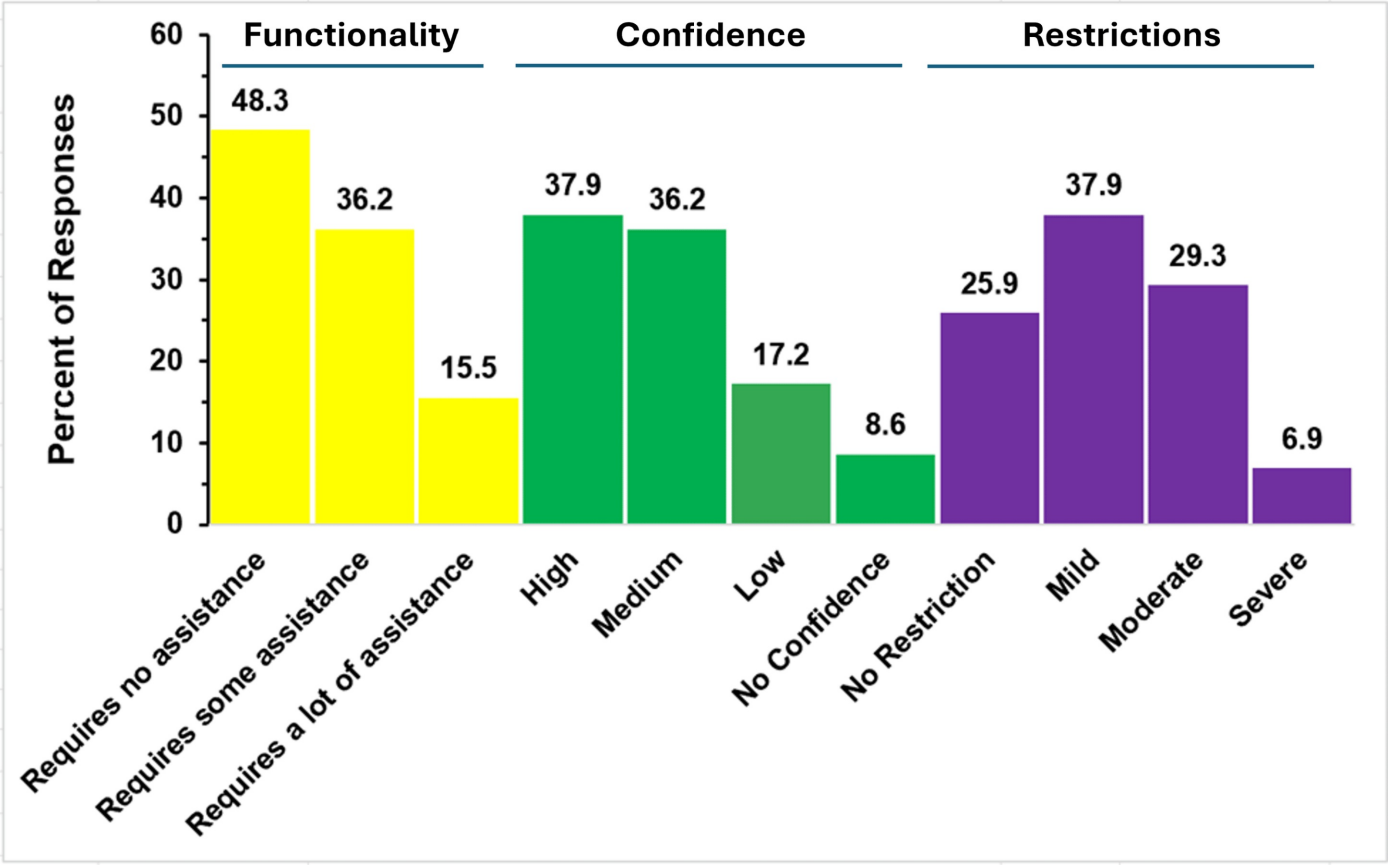
Functionality and restrictions in single ventricle The figure shows data from respondents who are parents of children with single ventricle. Percentages are based on the total number of single-ventricle respondents (N = 58) who answered the three questions regarding functionality, restrictions, and confidence that challenges may be reduced in the future.

A total of 58 responses from parents of children with a single ventricle were received and processed. Figure 13 summarizes their responses to questions about their child’s functionality and restrictions.

Functional Challenge

Regarding functional challenges, only nine of the 58 respondents (15.5%) reported that their child requires a lot of assistance. No assistance was cited most commonly, selected by nearly half of the respondents (28, 48.3%). The remainder of children (21, 36.2%) in this group need some degree of assistance.

Parents’ Confidence in Improvement

Parents' confidence in their child’s functional ability to improve was reported to be high in 37.9% of respondents (22 respondents), with a similar percentage (21, 36.2%) reporting medium confidence. Contrastingly, 26% of respondents (15 respondents) reported having low or no confidence.

Restrictions

Regarding activity restrictions, 63.8% of respondents (37 respondents) reported no restrictions or only mild restrictions with the remaining 36.2% (21 respondents) reporting moderate or severe restrictions. However, only 6.9% (four respondents) indicated that their child had severe restrictions.

Child Developmental and Physical Challenges

**Figure 14:**
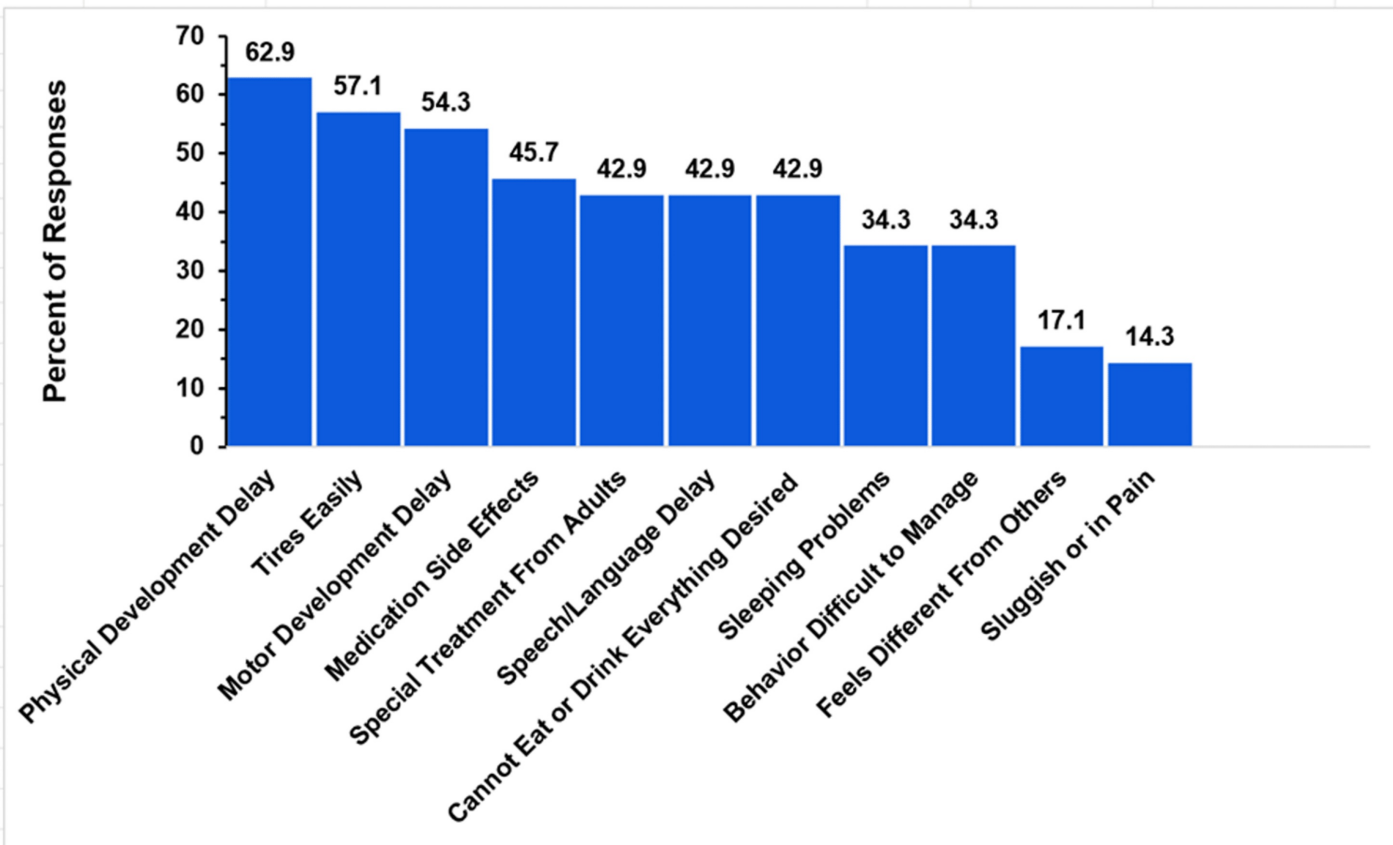
Single ventricle development delays and symptoms The figure shows data from respondents who are parents of children with single ventricle. Percentages are based on the total number of single-ventricle respondents who reported one of the physical symptoms or development delays listed (N = 35). Respondents could not select an “all of the above” option.

Figure 14 depicts the developmental and physical challenges faced by children with a single ventricle, as reported by their parents. Multiple choices could be chosen in response to this question, but there was no option to select “all of the above.”

Forty-one responded to this issue, with six indicating no physical or developmental challenges. The responses of the remaining 35 respondents are shown in Figure 14. The most common issue was physical developmental delay, noted in 62.9% of cases (22 respondents). This was followed by children tiring easily (20, 57.1%) and motor developmental delay (19, 54.3%). Medication side effects (16, 45.7%) were relatively frequent, and 42.9% (15 respondents) reported their child receiving special treatment from adults, experiencing speech or developmental delays, or being unable to eat or drink everything they desired. Sleep problems and behavior issues were reported by approximately one-third (12, 34.3%). Less frequently reported concerns included feeling different from others (six, 17.1%) and experiencing feelings of sluggishness or pain (five, 14.3%).

Caregiver Emotional Burden

**Figure 15:**
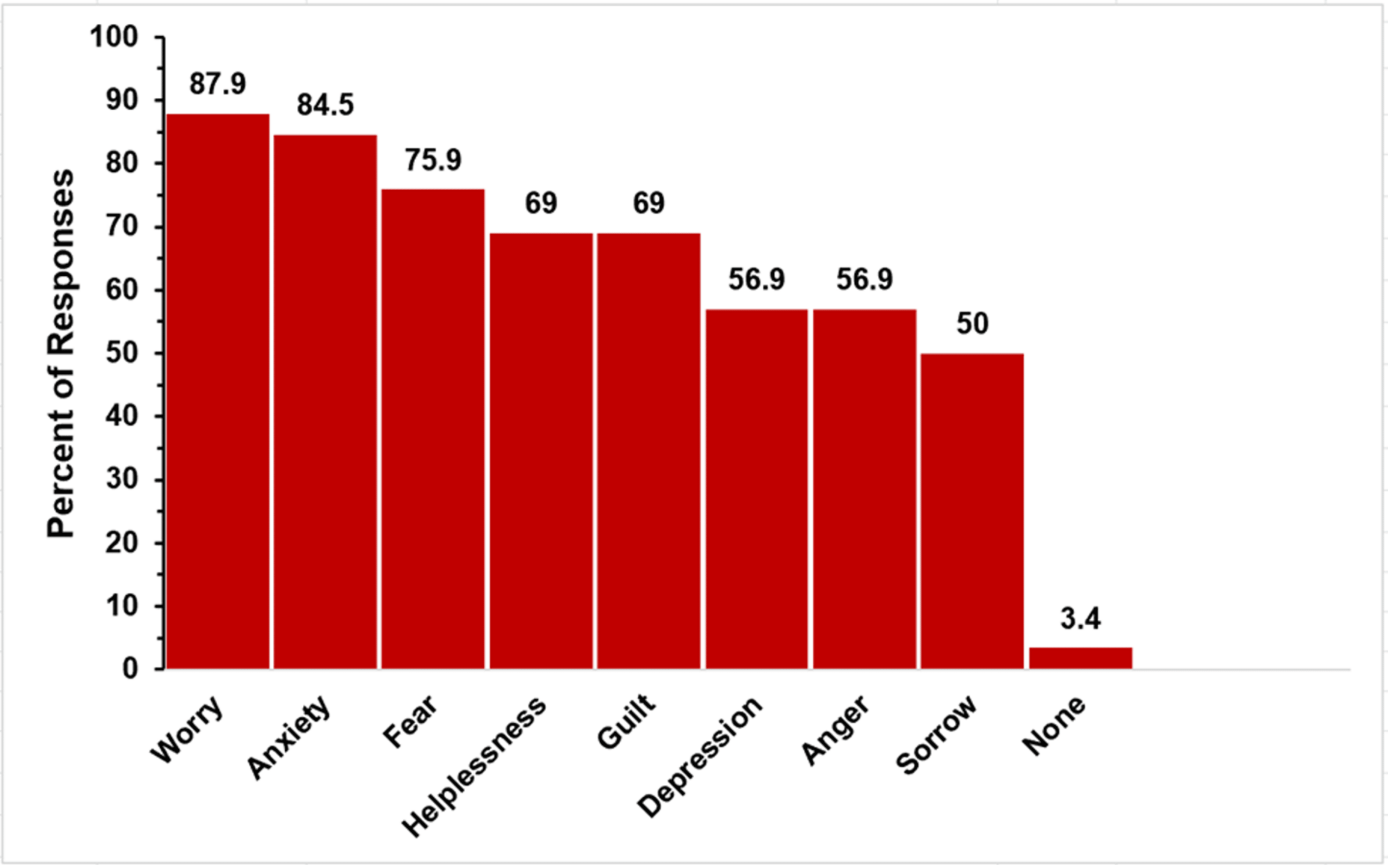
Parent feelings in single ventricle The figure shows data from respondents who are parents of children with single ventricle. Percentages are based on the total number of single-ventricle respondents (N = 58) who answered the question regarding feelings they have experienced as a product of their child’s condition. Respondents could not select an “all of the above” option.

The emotional burden on caregivers is depicted in Figure 15. Multiple choices could be chosen in response to this question, but there was no option to select “all of the above.”

Parents reported experiencing many troubling emotions in the single-ventricle group. Worry (51, 87.9%), anxiety (49, 84.5%), and fear (44, 75.9%) were the top three emotions. Furthermore, more than 50% of these parents reported feelings of helplessness, guilt, depression, anger, and sorrow. Only 3.4% (two respondents) reported having none of these emotions.

Access to Care and Economic Hardship of Caregivers

**Figure 16:**
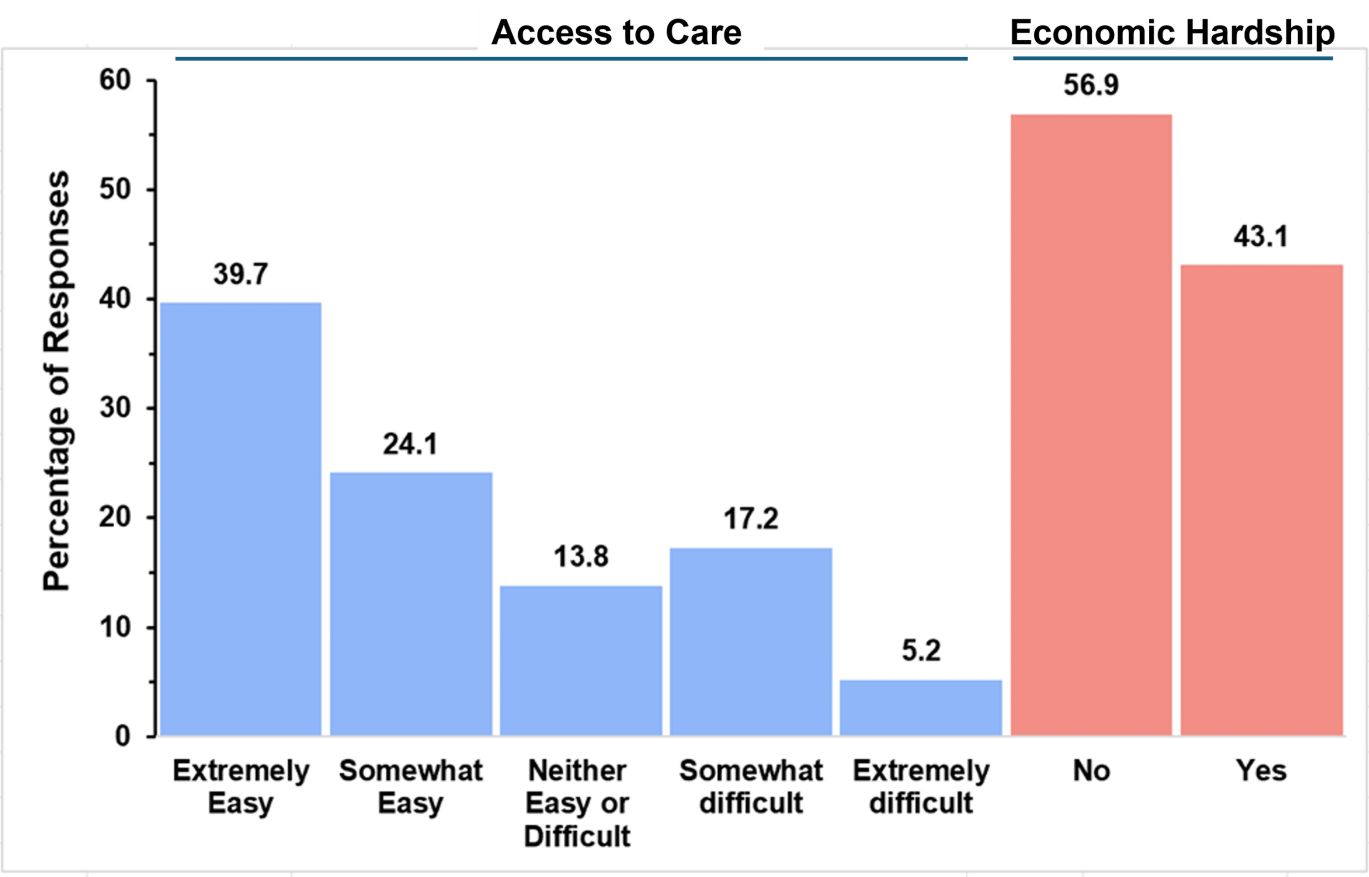
Healthcare access and financial burden in single ventricle The figure shows data from respondents who are parents of children with single ventricle. Percentages are based on the number of single-ventricle respondents (N = 58) who answered the questions regarding access to care and economic hardship.

Figure 16 graphically illustrates access to care and economic hardship in the single-ventricle group.

The majority of parents reported that accessing care was relatively easy, with 23 out of 58 parents (39.7%) reporting it as extremely easy and 14 respondents (24.1%) reporting it as somewhat easy. In contrast, 10 parents (17.2%) reported that access to care was somewhat difficult, while three respondents (5.2%) found it extremely difficult. The remaining eight respondents (13.8%) felt that access was neither easy nor difficult. Regarding financial strain, 25 out of 58 respondents (43.1%) reported experiencing economic hardship related to their child’s condition, while 33 respondents (56.9%) did not report financial difficulties.

Hospital Admissions

**Figure 17:**
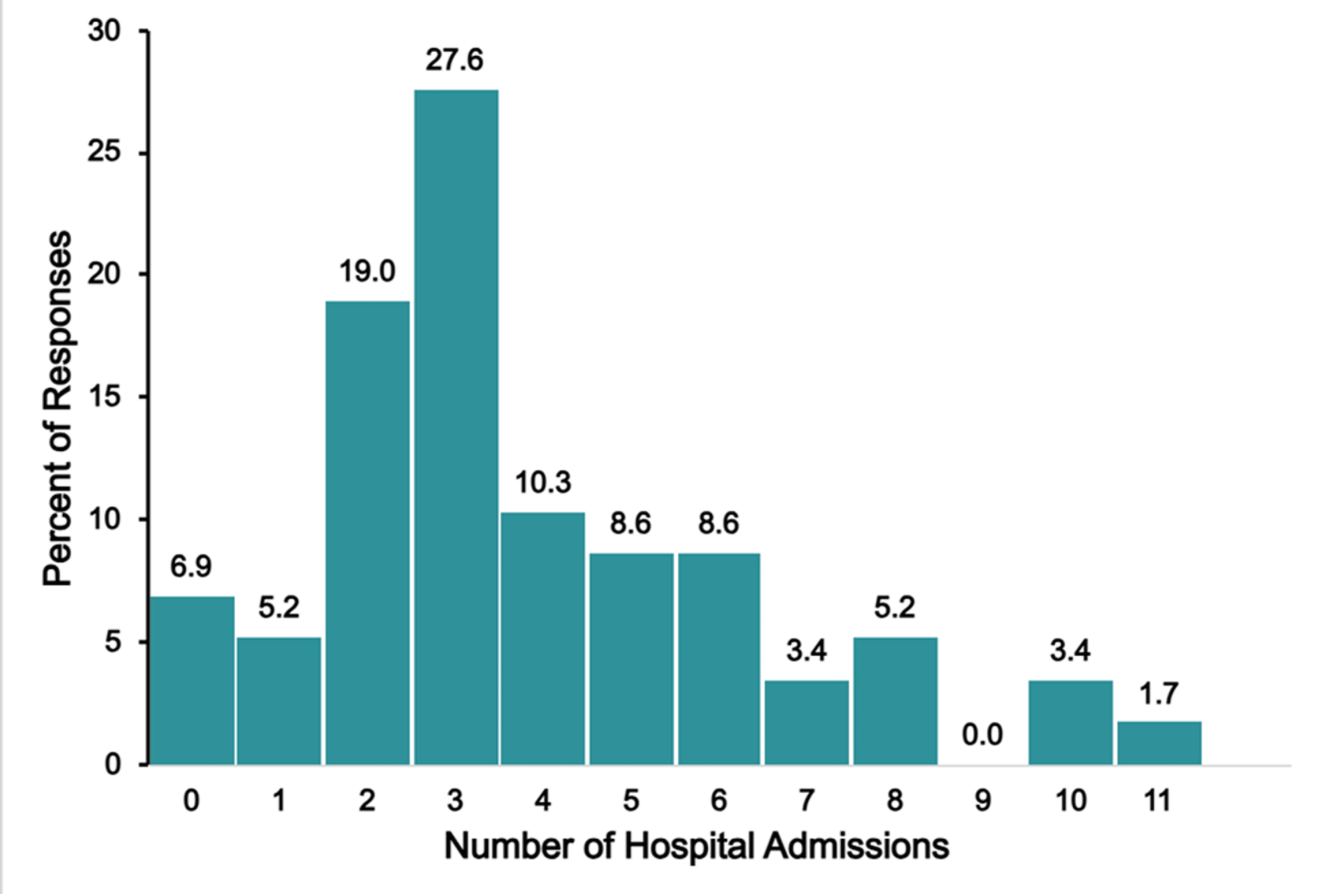
Single-ventricle hospitalizations The figure shows data from respondents who are parents of children with single ventricle. Percentages are based on the number of single-ventricle respondents (N = 58) who answered the question regarding the number of hospitalizations their child has needed.

Figure 17 shows the number of hospital admissions that single-ventricle respondents reported their child required due to the child’s congenital condition. 

**Figure 18:**
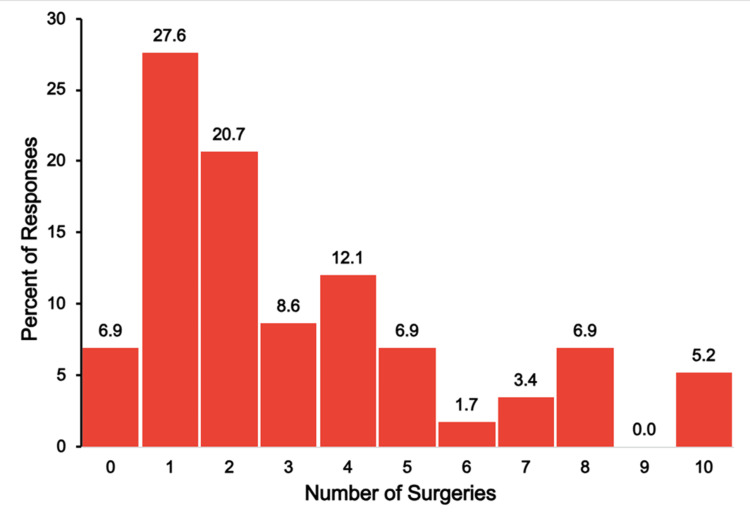
Single-ventricle surgeries The figure shows data from respondents who are parents of children with single ventricle. Percentages are based on the number of single-ventricle respondents (N = 58) who answered the question regarding the number of surgeries their child has needed.

Figure 18 shows the number of surgeries that single-ventricle respondents reported their child required due to the child’s congenital condition.

Hospitalization burden varied widely in this group, with 6.9% (four respondents) reporting no hospitalizations and 30.9% (18 respondents) reporting five or more hospitalizations. The most frequently reported number of hospitalizations was reported by 27.6% of respondents (16 respondents), and 5.1% (three respondents) indicated their child had experienced 10 or more hospitalizations for their congenital condition.

As with hospitalizations, the surgery burden varied widely in this group. Just a few (four, 6.9%) of respondents reported that their child had no surgeries related to their single-ventricle condition. The explanation for the absence of surgery, as reported, is unclear. The need of one or two surgeries was reported by nearly half of the respondents (28, 48.3%). However, approximately one-quarter of respondents reported the need for five or more surgeries (14, 24.1%) and 12.1% (seven respondents) reported their child needed eight or more surgeries.

Other Relevant Survey Data

Table 5 indicates additional findings as reported in the survey.

**Table 5:** Response distribution for remaining single-ventricle questions (N = 58) The response distribution for the following questions regarding single ventricle was not displayed in graphic format, but is included in table: Age at diagnosis, medical visit frequency, medications needed for condition, and age at first surgery. Percentages are based on the total number of single-ventricle respondents.

Age at Diagnosis	N (%)	Medical Visit Frequency	N (%)	Medications Needed for Condition	N (%)	Age at First Surgery	N (%)
Prenatal	45 (78%)	Daily	1 (2%)	0	4 (7%)	< 1 week	22 (38%)
< 1 week	8 (14%)	Weekly	3 (5%)	1	15 (26%)	1 week	8 (14%)
2 weeks	1 (2%)	Monthly	12 (21%)	2	13 (22%)	2 weeks	6 (10%)
3-5 months	3 (5%)	3 months	17 (29%)	3	5 (9%)	3 weeks	2 (3%)
Over 5 months	1 (2%)	6 months	16 (28%)	4	7 (12%)	1 month	8 (14%)
		Yearly	9 (16%)	5	4 (7%)	2 months	2 (3%)
				6	1 (2%)	3 months	1 (2%)
				7	2 (3%)	4-9 months	9 (16%)
				8	4 (7%)		
				10	3 (5%)		

The majority of children (45, 78%) in the single-ventricle group were diagnosed prenatally. If not diagnosed prenatally, diagnosis within one week of life was reported by 14% of respondents (eight respondents). Diagnosis any later than one week of life was reported by only 9% of respondents (five respondents). Medical visit frequency was highly variable. The most common responses were follow-up every three months, reported by 29% of respondents (17 respondents), followed closely by every six months, reported by 28% of respondents (16 respondents), and monthly visits, reported by 21% of respondents (12 respondents). Medication usage was generally low in the single-ventricle group, with four respondents (7%) reporting no medications at all, and more than half of the respondents (32, 55%) indicating that two or fewer medications were required for their child's congenital condition. All responders of the single-ventricle group underwent surgery, and most of them had it very early in life, with 62% of the children (36 respondents) having their first surgery within two weeks of age.

## Discussion

This paper aims to provide insight into the lived experiences of parents who have a child with a rare congenital heart condition. It examines the child's functional abilities, development, parents' emotional experiences, access to care, and financial burdens. These findings may help other parents understand what their child's journey might involve if they are diagnosed with such conditions.

Considerations for total anomalous pulmonary venous return

The survey results for total anomalous pulmonary venous return (TAPVR) were mostly positive; however, a wide range of experiences were reported, likely due to the different variants of this condition with varying outlooks. Most parents receive their child’s diagnosis within one month of birth, often within the first week, and sometimes even before birth. This condition can be life-threatening if not treated in the first few weeks of life [2], but with proper intervention, the survey shows that most children are likely to develop full, functional independence without restrictions. At least 60% of parents reported that their child requires no assistance and has no activity restrictions at all. Another positive point is that the risk of needing significant help (6.7%) or facing severe activity restrictions (2.2%) is very low. While parents should be prepared for their child to need some help and have certain activity restrictions, it is important to know that this diagnosis does not mean they cannot lead fully functional lives.

Based on the survey results, most children with TAPVR eventually become fully functional, but parents should be aware that developmental delays and general physical issues are common. Development delays are more likely to be a part of these families’ experiences than other symptoms. If development problems occur, parents can expect just over a 50% chance of delays in motor skills, speech, language, or physical abilities. Additionally, if other issues develop, parents can expect a 20%-35% chance that they are related to energy, sleep, diet, behavior, medication side effects, or the need for special accommodations. However, parents should also consider that very few survey respondents reported their child needing a lot of help in daily life or having severe restrictions, which suggests these issues are often manageable and rarely debilitating.

The survey results showed that parents of children with TAPVR should brace for an emotionally tough journey. Parenting is emotionally demanding no matter what, but parents of children with TAPVR are more likely to feel worried, anxiety, and fear about their child’s condition. Many will also face guilt, helplessness, depression, grief, or anger. While avoiding these feelings completely is impossible, being prepared can help families cope with this stressful experience. The survey suggests that seeking emotional counseling is helpful and should be encouraged for these parents.

Fortunately, most parents and children dealing with TAPVR will not face significant challenges accessing care. Access to medical resources often depends more on individual circumstances than the specific diagnosis itself. Nonetheless, parents in this situation can generally expect their child to receive care with relative ease. However, this care frequently involves a financial burden, which about half of the respondents in this survey reported. Financial difficulties in the US healthcare system are complex, and dealing with them will vary for each family. Nevertheless, families should consider this potential challenge early on to prepare effectively. Parents should understand that the financial strain is not unique to this diagnosis, as nearly half of US adults reported difficulty affording healthcare, according to a 2025 review [25].

In terms of medical experience, nearly all parents should be prepared for their child to undergo surgery within the first month of life, often before leaving the hospital after birth. Parents should also be aware that repeat surgeries are a common occurrence. Although the results indicate that only one out of 10 children required more than three surgeries, there is a potential for multiple surgeries, with up to 10 reported in this survey. Hospitalization followed a similar pattern, with most (about 85%) requiring two or fewer visits to manage their symptoms. However, some respondents reported up to 30 hospitalizations. Outpatient follow-up for these children is usually annual or biannual, and the medication burden was low in this group. Most reported needing no medication, and only 7% required more than three medications. In severe cases, outpatient follow-up may be weekly or monthly, and as many as 10 daily medications have been reported. In most cases, families dealing with TAPVR should not expect recurrent surgeries, hospital visits, and ongoing medical follow-up to interfere with the child's childhood, but they should be prepared for the possibility of this occurring in severe cases.

Considerations for Truncus Arteriosus

The survey responses about truncus arteriosus revealed some reassuring information for parents but also highlighted its potential challenges for children and families. A key factor to consider is its strong association (12%-35%) with DiGeorge syndrome [3], a chromosomal microdeletion that can cause many complications and different levels of severity. Whether or not this condition is present should be considered when thinking about what a child’s life might be like with truncus arteriosus. Still, the long-term outlook for truncus arteriosus is positive, with many patients eventually becoming asymptomatic [3]. The survey results support this.

Perhaps the most encouraging part of the survey responses for this condition was that 65% of respondents reported their child requires no assistance in daily living, and just 8% reported they need a lot of assistance. Additionally, 50% of respondents reported that their child has no activity restrictions, and among those who did, very few (2.9% of the total) had severe restrictions. This aligns with previous studies [9], which indicate a similar quality of life to that of age-matched controls, and should be a promising result for parents to consider when dealing with the early complications of this disorder.

The most common issues parents reported in this group involved developmental delays. Among those who noted their child’s problems, just over half mentioned physical or speech and language delays. Regarding physical symptoms, parents should be ready for their child to tire easily or face sleep or dietary challenges. Some children might also need special accommodation. However, only 31 of the 69 respondents (44.9%) answered this question, and nine reported no developmental delays or physical symptoms. Based on responses about daily functioning and activity restrictions, parents should remember that these symptoms and delays are often temporary or manageable. Still, parents of children with truncus arteriosus should be prepared for their child’s development to differ from that of others.

The survey responses also showed that parents of children with truncus arteriosus are likely to experience significant emotional distress. Worry, anxiety, and fear were reported almost universally. More than half of the respondents struggled with helplessness, guilt, depression, sorrow, and anger. While parents of healthy children might also often feel some of these emotions, they should recognize that they are especially at risk, and early psychological counseling is both appropriate and recommended.

An important positive finding was that parents did not face significant difficulty accessing care for their children with truncus arteriosus. Just 8.7% reported difficulty in accessing care, with only 2.9% reporting it was more than somewhat difficult. Economic hardship was common, but the majority reported no difficulty. However, parents should be aware that access and affordability of healthcare are complex, multifactorial issue that impacts many Americans with and without serious illness. The respondents also reported their overall healthcare burden, which demonstrates the major variance in outcomes for these children. Nearly all truncus arteriosus diagnoses are made prenatally or within the first week of life, and parents should expect that to be followed by immediate surgical repair. Fortunately, following primary surgical repair, the 20-year survival rate is over 80% [3]. However, parents should be aware that the need for reoperation is very likely, according to both documented surgical data [26] and the survey results. Over 75% of the respondents reported undergoing more than one surgery, with the majority (70%) reporting between two and five surgeries in total, and as many as 13 in one instance. Parents can expect a similar chance of recurrent hospitalization, with 68% requiring more than one total, and as many as 14 reported in one instance. Additionally, parents must be prepared for frequent outpatient follow-up, including serial echocardiography, with 75% of patients reporting monthly visits. Parents of children with truncus arteriosus would benefit from being educated about the significant healthcare burden their family is likely to face, in order to best prepare. Parents should also keep in mind that despite this long and arduous healthcare journey, their efforts have a strong chance of helping their child live a fully functional life.

Considerations for Single-Ventricle Defect

Single-ventricle defects exhibit a wide range of anatomical variations, resulting in diverse outcomes. The most common variants are hypoplastic left heart syndrome (HLHS), tricuspid atresia, and Ebstein's anomaly [27]. According to current research, HLHS, the most prevalent variant, has an 18-year survival rate of over 90% after it remains stable for the first year of life [27]. Survival rates for other variants may be lower, but these conditions are very rare, with limited long-term data, and most available outcome information is specific to individual centers [28]. The results discussed here can offer additional insight for parents of children with these conditions.

The most encouraging aspect of these results for parents is that nearly half of all respondents reported their child requires no assistance in daily living. In addition, out of those who required assistance, twice as many reported needing some assistance as opposed to a lot of assistance. However, parents should be prepared for their child to deal with activity restrictions, which most respondents reported (74%). Fortunately, the vast majority of restrictions were considered mild to moderate, with severe restrictions being uncommon.

Developmental delays and other physical symptoms were common in this group. A total of 41 respondents answered this question, with only six reporting that none of the options applied to their child. Among those who reported issues, more than half experienced tiring easily, delays in physical development, or motor delays. Over 40% reported medication side effects, dietary restrictions, speech or language delays, or the need for special accommodations. Parents of children with a single-ventricle defect should be aware of the high likelihood that their child will face a combination of these issues. Proper education on managing these problems will be essential for these families. Fortunately, based on responses about functionality, parents should understand that severe restrictions requiring extensive assistance are not common results of these issues.

Just two of the 58 parents reported that none of the emotions listed in the survey applied to their experience with single-ventricle defects. Therefore, parents would benefit from being educated on this emotional journey they will likely face. Emotional counseling and healthy stress outlets may be able to help combat the worry, anxiety, and fear that these parents frequently report. According to the results, most will also experience some feeling of helplessness, guilt, depression, anger, or sorrow. Early intervention, with as much social support as possible, is likely the best way to prepare to face this emotionally taxing time for parents.

A total of 13 out of 58 respondents reported difficulty in accessing care. Fortunately, only three of them found it to be extremely difficult. Most still said accessing care was easy, so parents can be reassured by these results. However, parents should also understand that access to care depends largely on individual circumstances and that healthcare access is a universal issue in the United States, not just for those with rare conditions. The same applies to financial burdens, as personal circumstances play a significant role, and it is a common problem for many Americans. While most in this group reported no financial hardships, parents should be aware that it can become an issue for many, so early planning is advisable.

The reported healthcare burden in the single-ventricle group was highly variable, reflecting the numerous variants of this condition and their varying circumstances. Parents should be educated about their child’s specific condition and what to expect from ongoing treatment. Most parents will be informed of their child’s diagnosis prenatally, and surgery within the first week to first month of life is the most likely outcome. Parents can expect repeat surgeries, which 65% of the respondents reported, with 25% requiring five or more. Repeat hospitalization was even more common (88%), 30% required five or more visits, and as many as 11 were reported in one instance. Outpatient follow-up varied, with almost everyone reporting follow-up intervals ranging from monthly to yearly. Parents should be informed as soon as possible that their child may require frequent and intensive medical attention. This will help parents be prepared to act when their child needs attention and help them brace for a potentially long journey.

Overall Considerations

Congenital heart conditions have a very wide range of outcomes and experiences for parents, varying between different types and subtypes of these conditions [29,30]. Overall, the data shows the potential for sustaining a high quality of life with minimal healthcare burden, while highlighting the possibility of having significant limitations and requiring high volumes of healthcare utilization. The data from this survey allows for some comparison between conditions, but parents should understand that many factors beyond the initial diagnosis influence their journey. Regarding the three conditions discussed, TAPVR showed the highest level of functionality, edging out truncus arteriosus slightly. On average, based on this survey's results, parents of children with single-ventricle defects can expect a greater likelihood that their child will need assistance with daily activities, face developmental issues or other symptoms, or encounter challenges accessing care compared to the other two conditions. TAPVR was associated with a significantly lower healthcare burden than the others, while truncus arteriosus and single-ventricle defects both required frequent surgeries and hospital stays. Emotional burden was reported nearly universally, regardless of the condition. Furthermore, it is documented that children with congenital heart diseases are at higher risk for mental health issues in adulthood [4]. The severity of these issues correlates directly with the patient’s functional status [5]. This emphasizes the need for parents of children with these conditions to receive proper education on early psychological support for both them and their child.

Limitations

Several potential limitations of this study should be considered. Although the questions posed in the survey were designed to elicit information in areas that the authors considered important, no independent validation procedure was subsequently used. The authors acknowledge that lack of a validated questionnaire is a major limitation of the study. It is encouraged for future research contributing to this topic to use validated instruments, but the goals of this study were achieved without validation of the survey. In addition, although the reported data were based on at least 50 participant responses, a larger sample size with collection of demographics would have been preferable. Due to the limited number of respondents, it was felt that stratifying results by demographic would not help draw any additional conclusions and was therefore excluded. The authors recognize this as a major limitation of the study. However, given that the study dealt with extremely rare conditions, this limitation may be unavoidable. Another potential limitation is that the data are self-reported, and for subjective questions, rely on the feelings recollected at the time of the survey responses. However, this is an intrinsic feature of survey-acquired data. Another limitation to consider is the survey was only distributed in English, which limits responses from those who are non-English speaking. In addition, the survey is limited to those who are apart of support groups, which may or may not represent the entire population of those dealing with rare congenital heart conditions. However, this is a limitation associated with very rare conditions, as the diversity of populations available for survey is limited.

## Conclusions

The survey results indicate that the experience of being a parent to a child with a rare congenital heart condition is often highly unpredictable. Within each of these conditions, there are numerous specific aspects of each variant that significantly impact outcomes and quality of life, resulting in dramatically different appearances. These results present a composite of responses from parents of children who fall under one of the three congenital cardiac anomalies, without specifying the subtype. They demonstrate that in most cases, children with these conditions will have easy access to care and often lead fully functional lives following the appropriate medical interventions. The results also show the potential for poor quality of life with a severe healthcare burden, which fortunately was a small minority, and much less common than those with minimal to mild disability. Emotional burden, however, was essentially unavoidable for these families, so early efforts to seek counseling and develop healthy coping skills are highly encouraged.
